# Acute effects of ferumoxytol on regulation of renal hemodynamics and oxygenation

**DOI:** 10.1038/srep29965

**Published:** 2016-07-20

**Authors:** Kathleen Cantow, Andreas Pohlmann, Bert Flemming, Fabienne Ferrara, Sonia Waiczies, Dirk Grosenick, Thoralf Niendorf, Erdmann Seeliger

**Affiliations:** 1 Institut für Vegetative Physiologie, Center for Cardiovascular Research, Charité–Universitätsmedizin Berlin, Campus Mitte, Berlin, Germany; 2 Berlin Ultrahigh Field Facility, Max Delbrück Center for Molecular Medicine in the Helmholtz Association, Berlin, Germany; 3 Physikalisch-Technische Bundesanstalt (PTB), Berlin, Germany

## Abstract

The superparamagnetic iron oxide nanoparticle ferumoxytol is increasingly used as intravascular contrast agent in magnetic resonance imaging (MRI). This study details the impact of ferumoxytol on regulation of renal hemodynamics and oxygenation. In 10 anesthetized rats, a single intravenous injection of isotonic saline (used as volume control) was followed by three consecutive injections of ferumoxytol to achieve cumulative doses of 6, 10, and 41 mg Fe/kg body mass. Arterial blood pressure, renal blood flow, renal cortical and medullary perfusion and oxygen tension were continuously measured. Regulation of renal hemodynamics and oxygenation was characterized by dedicated interventions: brief periods of suprarenal aortic occlusion, hypoxia, and hyperoxia. None of the three doses of ferumoxytol resulted in significant changes in any of the measured parameters as compared to saline. Ferumoxytol did not significantly alter regulation of renal hemodynamics and oxygenation as studied by aortic occlusion and hypoxia. The only significant effect of ferumoxytol at the highest dose was a blunting of the hyperoxia-induced increase in arterial pressure. Taken together, ferumoxytol has only marginal effects on the regulation of renal hemodynamics and oxygenation. This makes ferumoxytol a prime candidate as contrast agent for renal MRI including the assessment of renal blood volume fraction.

Ferumoxytol consists of iron oxide nanoparticles encapsulated by a polyglucose sorbitol carboxymethylether coating that is approved in the USA and the EU as intravascular iron supplementation therapy for patients with iron deficiency anemia related to chronic kidney disease (CKD). Since ferumoxytol is superparamagnetic, it is increasingly used off-label as contrast agent in magnetic resonance imaging (MRI) for a broad spectrum of preclinical and diagnostic imaging applications^1^,^2^,^3^,^4^,^5^,^6^,^7^,^8^,^9^. Ferumoxytol has been proven very useful for vascular MRI since it can be administered by intravenous injection and exhibits a long intravascular half-life of >14 hours in humans and about 2 hours in small rodents^1^,^2^,^7^. Applications range from angiography of various vascular beds including the renal vasculature to the assessment of the blood volume fraction^1^,^2^,^3^,^5^,^6^,^7^. The latter is of relevance for blood oxygenation level-dependent (BOLD) MRI, because the effective relaxation time T_2_* is linked to the amount of deoxygenated hemoglobin per tissue volume (voxel). BOLD MRI is increasingly utilized for the study of kidney disorders^10^,^11^,^12^,^13^,^14^. Since the impact of the renal blood volume fraction and its changes obviously exceed that of other organs such as the brain, assessment of the renal blood volume fraction is a prerequisite for unambiguous interpretation of renal BOLD MRI^10^,^11^.

The safety profile of ferumoxytol has mainly been assessed in anemic patients with CKD for whom ferumoxytol was originally approved^3^. A typical single dose in adult patients amounts to 510 mg Fe. The most serious adverse effect reported in the respective clinical trials was allergic responses; the FDA released warnings and changed prescribing instructions to decrease the risk of serious allergic reactions following intravenous iron replacement with ferumoxytol^15^. Hypotensive episodes were also observed in <2% of adult patients^1^,^3^.

The dose for ferumoxytol as off-label MR contrast agent is widely variable, according to literature reports, yet typically much lower than the dose used for therapeutic purposes^1^,^3^. Hypotensive episodes were also reported in three recent studies in children and young adults in whom ferumoxytol was used as MR contrast agent^16^,^17^,^18^. In one of these studies, serum creatinine and urea concentrations were measured and found unaltered by ferumoxytol^16^. Besides this single study, there are, to the best of our knowledge, no published reports on effects of ferumoxytol on the kidney. In particular, there are no data available with regard to its possible effects on renal hemodynamics and oxygenation. Such data are urgently needed, because, according to basic methodologic principles, it must be ascertained that a method intended to measure a certain variable does not *per se* alter this variable.

Realizing this lack of essential methodological data and recognizing the need of ferumoxytol-based monitoring of the renal blood volume fraction for a comprehensive assessment of renal hemodynamics and oxygenation, this work examines whether the substance itself may affect regulation of renal hemodynamics and oxygenation. To meet this goal, *in vivo* measurements of arterial blood pressure (ABP), total renal blood flow (RBF), local perfusion (laser-Doppler-flux) and tissue partial pressure of oxygen (pO_2_) in the renal cortex and medulla were employed^11^,^19^,^20^,^21^,^22^. Modulation of renal hemodynamics and oxygenation was accomplished by dedicated test interventions, including brief periods of aortic occlusion, hypoxia and hyperoxia^11^,^19^,^20^,^21^. Three (cumulative) doses of ferumoxytol were studied including a low dose regime based upon previous studies that monitored the blood volume fraction of the brain and kidneys in rats^6^,^7^ and a high dose regime that corresponds with the human equivalent dose (based upon body surface area)^23^ for the therapeutic use of ferumoxytol.

## Results

The surgical preparation and physiological experimentation to study regulation of renal hemodynamics and oxygenation was performed in 10 anesthetized rats, yet for anatomical and/or technical reasons, reliable data on RBF, cortical Laser-flux, and cortical pO_2_ could only be obtained in 9 rats, and data on medullary Laser-flux and medullary pO_2_ in 8 rats.

Prior to all interventions, the absolute baseline values for ABP, RBF, and tissue pO_2_ were recorded: ABP 98.3 ± 5.8 mmHg, RBF 4.86 ± 0.83 ml/min, cortical tissue pO_2_ 23.8 ± 3.2 mmHg, and medullary tissue pO_2_ 17.5 ± 3.0 mmHg. The laser-Doppler-flux-technique used to assess cortical and medullary perfusions allows to monitor relative changes yet does not provide absolute values^21^.

**Figure 1:**
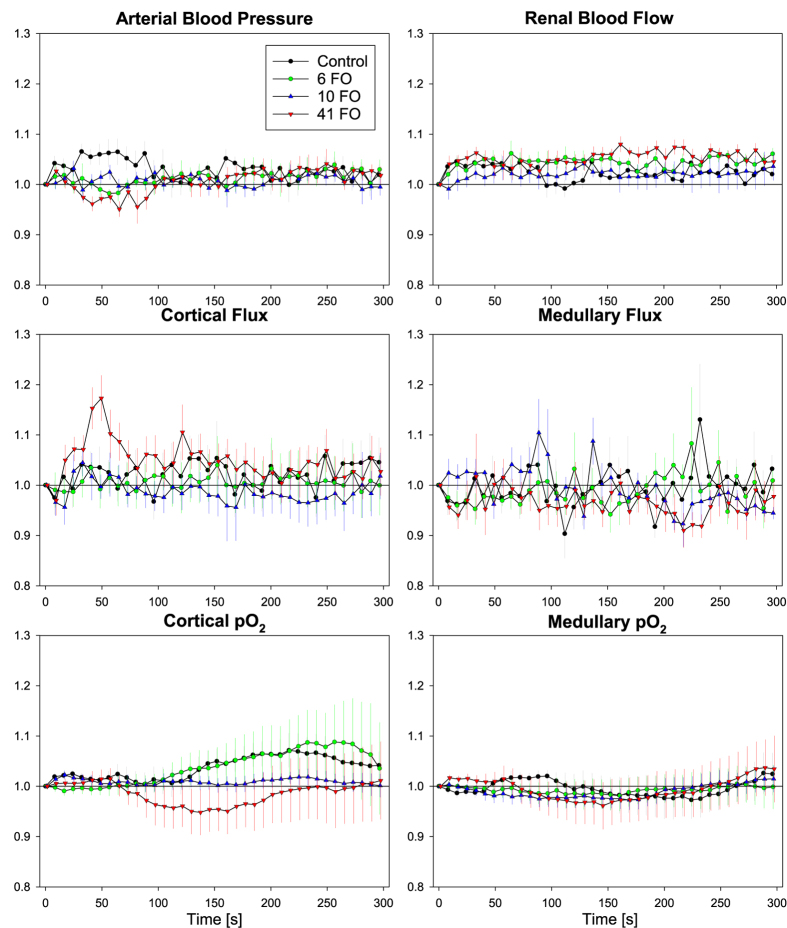
No sustained changes in physiological readings following ferumoxytol injections. Relative changes in arterial pressure, renal blood flow (transonic probe), cortical and medullary tissue perfusion (Flux, laser-Doppler-flux probes), and cortical and medullary tissue oxygen tension (pO_2_, fluorescence-quenching probes) upon injection of isotonic saline (volume Control), and three consecutive injections of ferumoxytol to achieve cumulative doses of 6, 10, and 41 mg Fe/kg body mass (6 FO, 10 FO, 41 FO). Data are mean ± SEM.

Figure 1 depicts the time course for all physiological parameters upon injection of saline (control) and upon injection of three cumulative doses of ferumoxytol. Apart from minor short-lasting fluctuations, the saline injection did not result in sustained changes in any of the physiological parameters under investigation. Likewise, none of the three injections of ferumoxytol resulted in marked sustained changes in any parameter, and there were no significant changes as compared to saline.

**Figure 2:**
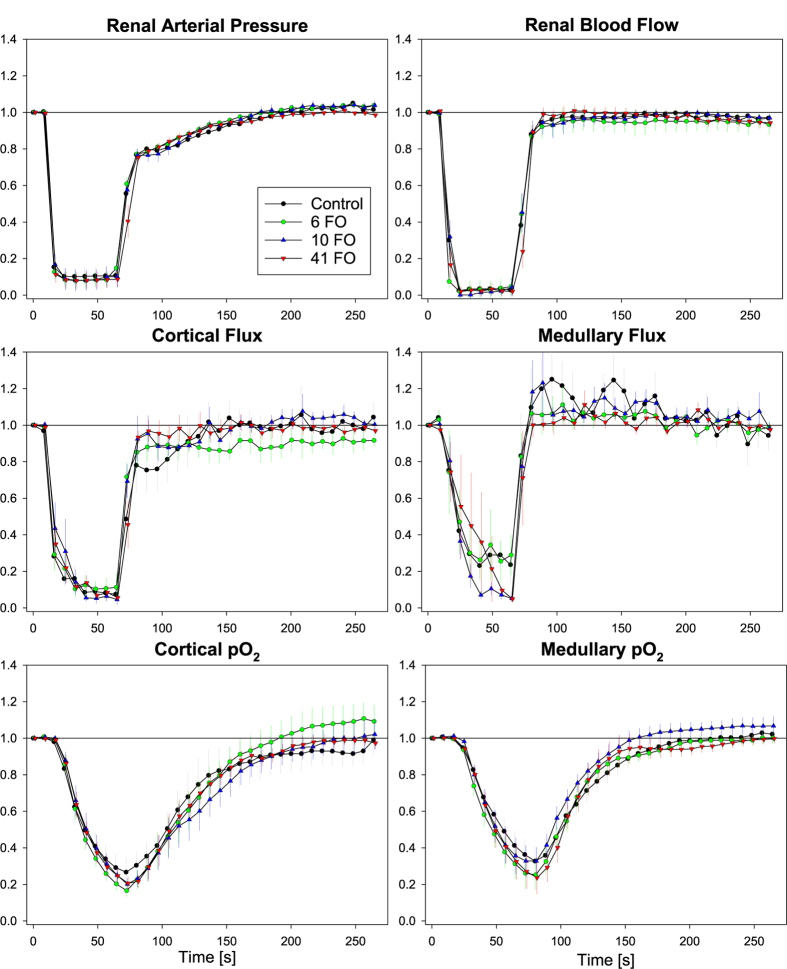
Ferumoxytol does not influence physiological changes during suprarenal aortic occlusion and recovery. Relative changes in renal arterial pressure, renal blood flow, cortical and medullary tissue perfusion (Flux), and cortical and medullary tissue oxygen tension (pO_2_) upon suprarenal aortic occlusion for 60 sec followed by restoration of the perfusion at cumulative ferumoxytol doses of 0 (Control), 6, 10, and 41 mg Fe/kg body mass (6 FO, 10 FO, 41 FO). Data are mean ± SEM.

Figure 2 illustrates the time courses during suprarenal aortic occlusion and recovery. Under control conditions, the inflation of the occluder resulted in an immediate drop in renal arterial pressure (RAP). Similarly, RBF approached zero instantaneously upon onset of the occlusion; the fall in cortical and medullary fluxes was less abrupt. Cortical and medullary pO_2_ started to decrease shortly thereafter and reached nadirs only upon the release of the occlusion, after 60 sec. In response to the deflation of the occluder, RAP rapidly increased to about 80% of baseline followed by a gradual approach to baseline within 120 sec. RBF and the fluxes increased rapidly and approached baseline earlier than RAP. Cortical and medullary pO_2_ increased more slowly and reached their respective baseline values 150 and 120 sec after the release of the occlusion. None of the three doses of ferumoxytol resulted in significant changes in any of the physiological parameters measured, as compared to saline.

**Figure 3:**
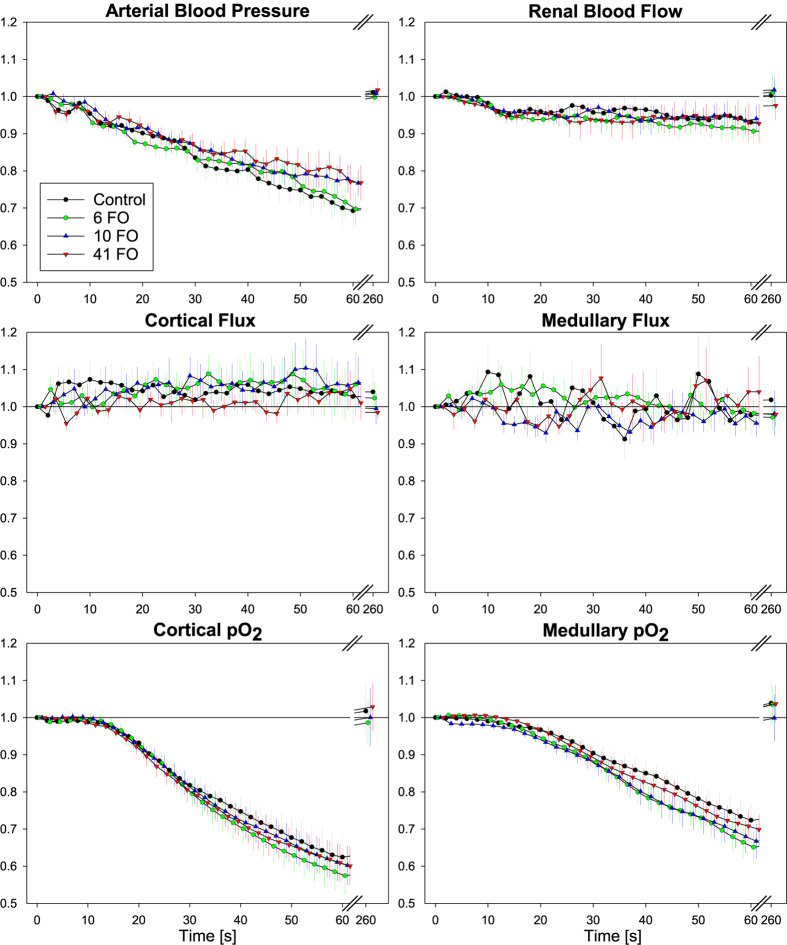
No changes in physiological parameters by ferumoxytol during hypoxic challenge. Relative changes in arterial blood pressure, renal blood flow, cortical and medullary tissue perfusion (Flux), and cortical and medullary tissue oxygen tension (pO_2_) upon a hypoxic challenge (inspiratory oxygen 10%) at cumulative ferumoxytol doses of 0 (Control), 6, 10, and 41 mg Fe/kg body mass (6 FO, 10 FO, 41 FO). Data at time 260 sec indicate that all variables had returned to baseline values at the end of the respective recovery period (200 sec of normoxia). Data are mean ± SEM.

Figure 3 surveys the time courses obtained in response to the hypoxic stimulus with an inspiratory fraction of oxygen (FiO_2_) of 10%. Under control conditions, ABP decreased continuously during the 60 sec of hypoxia, reaching about 70% of baseline. RBF decreased to a much lesser extent; cortical flux showed a small increase and medullary flux showed some fluctuations only. Cortical and medullary pO_2_ decreased continuously, reaching about 65% and 75% of their respective baseline values toward the end of the hypoxic period. With the restoration of normoxia (FiO_2_ 21%), all parameters returned to their respective baseline values within the recovery period of 200 sec. Similarly to the aortic occlusion and recovery intervention, none of the three doses of ferumoxytol resulted in any significant changes in all measured physiological parameters, as compared to the saline reference.

**Figure 4:**
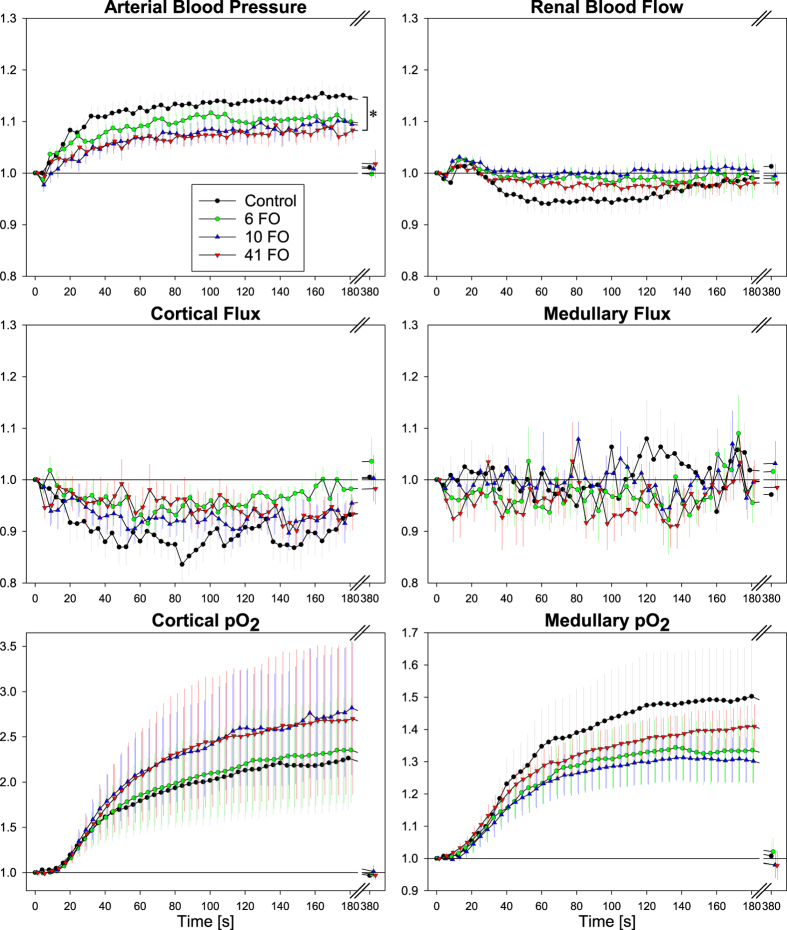
Ferumoxytol blunts ABP increase during a hyperoxic challenge. Relative changes in arterial blood pressure, renal blood flow, cortical and medullary tissue perfusion (Flux), and cortical and medullary tissue oxygen tension (pO_2_) upon a hyperoxic challenge (inspiratory oxygen 100%) at cumulative ferumoxytol doses of 0 (Control), 6, 10, and 41 mg Fe/kg body mass (6 FO, 10 FO, 41 FO). Data at time 380 sec indicate that all variables had returned to baseline values at the end of the respective recovery period (200 sec of normoxia). Data are mean ± SEM; *denotes significant difference (ANOVA for repeated measurements followed by Bonferroni’s multiple comparison procedure).

Figure 4 shows the time courses obtained in response to hyperoxia (FiO_2_ 100%). Under control conditions, ABP increased during the 180 sec of hyperoxia, reaching about 16% above baseline. Interestingly, ferumoxytol tended to blunt the hyperoxia-induced increase in ABP in a dose-dependent manner. At the highest dose, the pressure rise was significantly lower as compared to saline. On the other hand, none of the three doses of ferumoxytol resulted in significant changes in any other physiological parameter as compared to saline. Under both control and ferumoxytol conditions, RBF and cortical flux showed a small insignificant decrease and medullary flux some fluctuations. Even in cortical and medullary pO_2_, which increased continuously (reaching values about 2.2-fold and 1.5-fold their respective baseline values) over the hyperoxic time-frame, there were no significant changes during ferumoxytol treatment. With the restoration of normoxia (FiO_2_ 21%), all parameters returned to their respective baseline values within the recovery period of 200 sec.

## Discussion

This work adds to the literature by exploring the potential acute *in vivo* effects of ferumoxytol on renal hemodynamics and oxygenation. Our main finding is that intravenously injected ferumoxytol has only a marginal impact on the regulation of renal hemodynamics and oxygenation in healthy rats. Interestingly, the only significant effect observed was a blunting of the hyperoxia-induced increase in arterial pressure at a cumulative dose of 41 mg Fe/kg, a high dose that corresponds with the human equivalent dose for iron supplementation. Our results clearly show no immediate risk of impaired renal oxygenation or perfusion inherent to an injection of ferumoxytol, thereby rendering ferumoxytol a favourable contrast medium suitable for the quantification and monitoring of changes in renal blood volume fraction.

Regulation of renal hemodynamics and oxygenation was studied with periods of aortic occlusion, hypoxia, and hyperoxia. The responses to these interventions are brought about by complex interactions of renal and extrarenal mechanisms. Occlusion of the suprarenal aorta results in abrupt cessation of blood flow into the kidney. As renal O_2_ consumption initially remains unaltered, renal tissue pO_2_ declines rapidly. This leads to intrarenal vasodilation, which is augmented by accumulation of CO_2_ as well as by the mechanisms of autoregulation of RBF^20^,^24^,^25^. Vasodilation also takes place in the extrarenal tissues downstream of the occluder. By deflating the occluder, the kidney and the extrarenal tissues are reperfused. Due to the initially sustained vasodilation, systemic arterial pressure drops^25^. This is detected by arterial baroreceptors and actuates the baroreflex response which results in renal and extrarenal vasoconstriction. Again, mechanisms of RBF autoregulation contribute to the compensatory renal vasoconstriction^25^. That ferumoxytol did not alter the complex response to aortic occlusion and recovery (Fig. 2) indicates that all elements contributing to this response were left unaffected by the compound.

Likewise, ferumoxytol did not affect the mechanisms that govern the responses to hypoxia (Fig. 3). Switching the inspiratory oxygen fraction to 10% results in moderate arterial hypoxemia so that tissue pO_2_ in all vascular beds gradually decreases. Of course, renal tissue hypoxia is by far milder than during aortic occlusion. The ensuing systemic and renal vasodilation triggers counteraction by the baroreflex and the mechanisms of RBF autoregulation. In addition, the systemic hypoxemia is detected by arterial chemoreceptors that actuate a reflex response that increases ventilation. This results in lower rather than higher pCO_2_ which also counteracts vasodilation^24^,^25^,^26^.

Ferumoxytol did not significantly affect the responses to hyperoxia, with the exception of arterial pressure at the highest ferumoxytol dose (Fig. 4). The hyperoxia-induced pressure increase is due to vasoconstriction, preferentially in extrarenal vascular beds^27^,^28^. It is presumed to largely rely on direct impact of hyperoxia on the microvasculature, where increased pO_2_, probably *via* reactive oxygen species, can induce an imbalance of vasoactive factors including nitric oxide (NO), endothelin, and prostaglandins^29^,^30^. The mechanism(s) by which ferumoxytol blunted this response are completely unknown. One could hypothesize that ferumoxytol particles may increase the shear stress in the erythrocyte-free plasma cuff layer in the microvasculature, whereby it might counteract the hyperoxia-induced decrease in endothelial NO production. Taken together, our results indicate that the influence of ferumoxytol on regulation of renal hemodynamics and oxygenation in rats is negligible and make it an ideal candidate for MR-based assessment of the renal blood volume fraction.

Renal tissue hypoperfusion and hypoxia are considered to be key elements in the pathophysiology of acute kidney injury and its possible progression to CKD^31^,^32^,^33^,^34^. Because BOLD MRI offers a noninvasive technique to obtain insight into renal oxygenation, it is increasingly leveraged for the study of acute and chronic kidney diseases and its swift translation into the clinical realm^10^,^11^,^12^,^13^,^14^. Yet, the blood volume fraction is a major confounder of the relationship between T_2_* and tissue pO_2_ 10,11. For a number of reasons, the impact of the kidney’s blood volume fraction is greater than that of other organs. As compared per tissue weight, total renal blood flow is much higher than that of most other tissues, thus renal vasoconstrictive or vasodilatory effects elicited by neuronal, endocrine, paracrine, and metabolic factors as well as by the mechanisms of RBF autoregulation will probably result in larger changes^10^,^21^,^32^,^35^. In fact, substantial changes in the renal blood volume fraction were reported for a number of experimental settings^7^,^11^,^20^. Moreover, the tubules are a unique feature of the kidney. Their volume fraction is quite large and can rapidly change due to changes in glomerular filtration, alterations in tubular outflow towards the pelvis, changes in tubular fluid resorption, and modulation of the transmural pressure gradient. As the renal capsule is rather tough, changes in the tubular volume fraction will often result in circular distension or compression of intrarenal vessels, which will inevitably change the blood volume fraction^10^. The added complexity may very well confound interpretation of BOLD-MRI obtained from patients who suffer from renal disorders^35^,^36^,^37^,^38^. Thus, assessment of the renal blood volume fraction and its changes under various (patho-)physiological conditions is mandatory for an unambiguous interpretation of renal BOLD-MRI.

The use of ferumoxytol is conceptually appealing for the pursuit of MR-based assessment/monitoring of the renal blood volume fraction. Like other ultrasmall superparamagnetic iron oxide (USPIO) particles, ferumoxytol does not readily extravasate and is not filtered in the glomeruli, so it has a sufficiently long plasma half-life (about 2 h in rats; about 15 h in humans)^1^,^2^,^7^. Unlike other USPIOs, ferumoxytol has the advantage that it is approved for intravenous injection in humans–although it is, of course, off-label when used as contrast agent. The magnetite core of ferumoxytol is covered by a coating of polyglucose sorbitol carboxymethylether that hinders contact of the iron with blood plasma, thus allowing relatively fast administration. The commercially available solution (Feraheme) has an average particle size of 23 nm with a narrow particle size distribution^6^. The rather slow elimination of ferumoxytol from the blood pool occurs via phagocytosis by macrophages of the reticuloendothelial system^1^,^2^,^3^,^7^.

Ferumoxytol’s feasibility to monitor pharmacologically induced acute changes in renal blood volume fraction in rats has already been shown^7^. Our present study can be conceived as having accomplished the next mandatory step, i.e., to ascertain that ferumoxytol does itself not unduly affect regulation of renal hemodynamics and oxygenation in healthy rats. Whether this also applies to animals with renal disorders remains to be tested. The further experimental stage would be to determine the impact of changes in the blood volume fraction on the relationship between renal T_2_* and tissue pO_2_ under various (patho-)physiological conditions. This would ideally be done by use of an integrated approach such as MR-PHYSIOL that allows simultaneous monitoring of invasive physiological parameters and MR parameters in combination with *in vivo* dedicated test interventions of renal hemodynamics and oxygenation^11^,^21^. Of course, the impact of shifts in the oxyHb dissociation curve and changes in hematocrit on the relation between renal tissue pO_2_ and renal BOLD also remains to be determined^10^.

Our study has a number of limitations. First, we studied the effects of ferumoxytol on regulation of renal hemodynamics and oxygenation in anesthetized healthy rats. We did not observe any decrease in arterial blood pressure. This is not in line with previous reports on clinical studies that enrolled patients suffering from CKD-related anemia who received therapeutic doses (hypotensive effects in <2% of patients) as well as studies where patients were administered ferumoxytol off-label as MR contrast (incidence of hypotensive episodes 0.4 to 12%)^1^,^3^,^16^,^17^,^18^. Reasons behind this difference may include species differences and pre-existing disorders in the patients enrolled. Second, our study focussed on kidney hemodynamics and oxygenation so that the results may not apply to other organs or vascular beds. Finally, we chose two dosages of ferumoxytol based upon previous MR-studies on the blood volume fraction of the brain and kidneys in rats^6^,^7^. In addition, we studied the effects of the very high dose (41 mg Fe/kg) that accords with the human equivalent dose for iron supplementation. Human equivalent doses that are based on body surface area are required by authorities for determining the drug safety profile^23^. Thus, our results indicate a rather broad safety margin when typical ferumoxytol doses for MR contrast are applied. We are quite confident that even lower doses than our lowest dose (6 mg Fe/kg of BM) will suffice to study the impact of renal blood volume fraction on BOLD-MRI in rats.

To conclude, this study demonstrates the absence of any adverse acute effects of ferumoxytol on the regulation of renal hemodynamics and oxygenation. This finding renders ferumoxytol a prime candidate for intravascular contrast agent-based assessment of renal blood volume fraction without confounding renal oxygenation or perfusion.

## Materials and Methods

### Animal Preparation

The study was approved by the Animal Welfare Administration of Berlin’s State Office of Health and Social Affairs in accordance with the German Animal Protection Law and the experiments were carried out in accordance with the approved guidelines. Experiments including all interventions were performed in ten male Wistar rats (250–350 g BM; Harlan-Winkelmann, Borchen, Germany). The animals received food and water ad libitum and were housed under standard conditions with environmental enrichment. For anesthesia, urethane (20% in distilled water; 6 ml/kg BM intraperitoneal; Sigma-Aldrich, Steinheim, Germany) was used throughout the surgical preparation and the examination. This approach provides anesthesia for several hours and leaves cardiovascular and respiratory reflexes largely undisturbed. The rats were positioned on a thermostated table in order to maintain their body temperature at 37 °C.

A tracheal cannula was inserted to facilitate spontaneous breathing. Monitoring of absolute arterial blood pressure (in mmHg) that corresponds with renal arterial pressure (RAP) was achieved by placing a catheter into the femoral artery with its tip towards the aorta. The catheter was connected to a pressure transducer (DT-XX, Viggo-Spectramed, Swindon, UK) and amplifier (TAM-A Plugsys Transducer; Hugo Sachs Elektronik–Harvard Apparatus GmbH, March-Hugstetten, Germany). The femoral artery catheter also served for continuous saline infusion (1 ml/h) throughout the preparation and the experiment. Another catheter was inserted into the right jugular vein; this was used for subsequent injections of isotonic saline and ferumoxytol (see below).

The abdomen was opened by a midventral incision; during surgery and examination the abdominal cavity was filled with isotonic saline (37 °C). Monitoring of total renal blood flow was achieved by a flow probe (1RB, Transonic Systems, Ithaca, NY, USA) positioned around the left renal artery. This blood flow measurement relies on ultrasound transit time difference and thus provides absolute data (in ml/min). Monitoring of tissue pO_2_ and perfusion was enabled by two combined pO_2_/laser-Doppler-flux probes (pO_2_ E-Series Sensor; Oxford Optronics, Oxford, UK) that were inserted into the renal tissue, one into the medulla (about 4 mm from the renal capsule) the other one into the cortex (about 1.5 mm). The fluorescence-quenching optodes allow absolute measurements of tissue pO_2_ (in mmHg). The laser-Doppler-flux probes assess tissue perfusion by erythrocyte flux (arbitrary units), thus, only relative changes in perfusion are monitored. For induction of aortic occlusions a remotely operated inflatable cuff was positioned around the aorta above the renal arteries. All parameters were continuously monitored and, following analog-to-digital conversion, the data were stored online with a sampling rate of 50 Hz.

### Experimental Protocol and *in vivo* Interventions

Subsequent to the preparation, the experiments were started by recording baseline data of all parameters for 300 sec. The experimental protocol comprised four consecutive intravenous injections (after isotonic saline that served as volume control, three injections of ferumoxytol followed to achieve cumulative doses of 6, 10, and 41 mg Fe/kg BM, respectively) at intervals of about 23 min. During the intervals, the response of all variables to the injections was recorded, followed by the test procedures to characterize regulation of renal hemodynamics and oxygenation under the respective conditions.

The reason we choose the cumulative escalating dosage procedure was as follows: The study’s goal was to test the working hypothesis that ferumoxytol is not suitable as MR contrast agent for renal studies because it affects regulation of renal hemodynamics/oxygenation. Thus, ferumoxytol’s effects had to be compared with the volume control, while comparisons among the doses of ferumoxytol are needless. The goal to test the working hypothesis had to be achieved with as small a number of animals as possible, as stipulated by the German Animal Protection Law. The interval of about 23 min between injections was chosen to balance two competing constraints: on one hand, the interval should be as short as possible such that the plasma concentration of ferumoxytol remains nearly constant between injections (plasma half-live time of about 2 hours). On the other hand, the test procedures (aortic occlusion, hypoxia, hyperoxia) consume time, not the least because we had to allow ample time for full recovery following each intervention.

Each injection was preceded by a recording of baseline data. The injections amounted to 1 ml/kg BM, and were slowly administered (about 45 sec) into the jugular vein. The small volume and the rather slow injection rate were chosen in order to prevent that some kind of adaptation to a “volume expansion” may occur. In order to administer the same volume of saline and of each of the three doses of ferumoxytol, Feraheme (AMAG Pharmaceuticals, Inc., Waltham, MA, USA) was diluted with isotonic saline for the two lower doses and was used undiluted at the highest dose. The first dose of ferumoxytol contained 6 mg Fe/kg BM, the second dose contained 4 mg Fe/kg BM to achieve a cumulative dose of 10 mg Fe/kg BM, and the third dose contained 31 mg Fe/kg BM to achieve a cumulative dose of 41 mg Fe/kg BM. The response to the injection of saline and the three doses of ferumoxytol, respectively, was recorded for 300 sec post injection (results shown by Fig. 1).

In the ongoing intervals between the injections, the tests used to characterize regulation of renal hemodynamics and oxygenation were then performed. This comprised suprarenal aortic occlusion, hypoxia, and hyperoxia, each preceded by recording of baseline data and followed by a recovery period. Aortic occlusion was initiated by inflating the suprarenal aortic occluder, lasted for 60 sec, and was followed by 200 sec of recovery (results shown by Fig. 2). Hypoxia was induced by decreasing FiO_2_ from 21% (room air) to 10% *via* changing the gas flow to the tracheal cannula to 10% O_2_/90% N_2_ for 60 sec (results shown by Fig. 3), then, FiO_2_ was restored to 21%, and 200 sec allowed for recovery. FiO_2_ was monitored using a Capnomac AGM-103 (Datex GE, Chalfont St. Gils, UK). Hyperoxia was induced by increasing FiO_2_ from 21% to 100% *via* changing the gas flow to pure oxygen for 180 sec (results shown by Fig. 4), then, FiO_2_ was restored to 21%, and 200 sec allowed for recovery.

### Statistical Analysis

Relative values were obtained by relating the absolute data after initiating a given intervention to the absolute data obtained immediately before this intervention. All data are given as mean ± SEM. Statistical analyses were done by analysis of variance (ANOVA) for repeated measurements, followed by the Bonferroni’s multiple comparison procedure with a significance level of P < 0.05 using Number Cruncher Statistical Software (Hintze, Kaysville, UT, USA).

## Additional Information

**How to cite this article**: Cantow, K. *et al*. Acute effects of ferumoxytol on regulation of renal hemodynamics and oxygenation. *Sci. Rep.* **6**, 29965; doi: 10.1038/srep29965 (2016).
